# Mid-term Patency of the Great Saphenous Bypass to Aorta vs. Non-aortic Arteries in Stanford Type A Aortic Dissection Surgery With Concomitant CABG

**DOI:** 10.3389/fcvm.2021.743562

**Published:** 2021-10-26

**Authors:** Maozhou Wang, Songhao Jia, Xin Pu, Lizhong Sun, Ming Gong, Hongjia Zhang

**Affiliations:** ^1^Department of Cardiac Surgery, Beijing Anzhen Hospital, Capital Medical University, Beijing, China; ^2^Department of Interventional Therapy, Beijing Anzhen Hospital, Capital Medical University, Beijing, China

**Keywords:** patency, proximal target artery, Stanford type A aortic dissection, coronary artery bypass grafting, great saphenous vein

## Abstract

**Background:** Stanford type A aortic dissection (STAAD) is often associated with coronary artery problems requiring coronary artery bypass grafting (CABG). However, the prognosis of different proximal graft locations remains unclear.

**Methods:** From May 2015 to April 2020, 62 patients with acute STAAD who underwent aortic surgery concomitant with CABG were enrolled in our study. Aortic bypass was defined as connecting the proximal end of the vein bridge to the artificial aorta (SVG-AO); non-aortic bypass was defined as connecting the proximal end of the vein bridge to a non-aorta vessel, including left subclavian artery, left common carotid artery, and right brachiocephalic artery (non-SVG-AO). We compared early- and mid-term results between patients in the above two groups. Early results included death and bleeding, and mid-term results graft patency, aortic-related events, and bleeding. Grafts were evaluated by post-operative coronary computed tomography angiography. According to the Fitzgibbon classification, grade A (graft stenosis <50%) is considered a patent graft. Univariate and multivariate analyses were performed to assess differences between aortic and non-aortic bypass in STAAD.

**Results:** SVG-AO and non-SVG-AO were performed in 15 and 47 patients, respectively. There was no significant difference in death (log-rank test, *p* = 0.426) or bleeding (*p* = 0.766) between the two groups in the short term. One year of follow-up was completed in 37 patients (eight in the SVG-AO group and 29 in the non-SVG-AO group), among which 14/15 (93.3%) grafts were patent in the SVG-AO group and 32/33 (97.0%) grafts in the non-SVG-AO at 1 week, without a significant difference (*p* = 0.532). At 3 months, 12/13 (92.3%) grafts were patent in the SVG-AO group and 16/32 (50.0%) grafts in the non-SVG-AO, with a significant difference (*p* = 0.015), and 12/13 (92.3%) grafts in the SVG-AO group and 15/32 (46.9%) grafts in the non-SVG-AO group were patents, with a significant difference. Multivariate analysis showed proximal aortic bypass and dual anticoagulation to be protective factors for the 1-year patency of grafts.

**Conclusion:** In patients requiring aortic dissection surgery with concomitant CABG, no differencess' between SVG-AO and SVG-non-AO in early outcomes were detected, but SVG-AO may have higher mid-term patency.

## Introduction

Some patients with Stanford type A aortic dissection (STAAD) may have coronary artery problems, such as coronary artery involvement or coronary atherosclerotic heart disease (1–3). In addition, some patients with myocardial ischaemia or even myocardial infarction show significantly increased early mortality (3, 4). For patients with type C or some type B based on Nari's classification, coronary artery bypass grafting (CABG) is needed to improve coronary malperfusion (3).

Due to its urgency, the great saphenous vein graft (SVG) is the main graft for CABG in STAAD because of its convenience and rapid access (5). At present, there are two main types of proximal artery bypass grafts after ascending and total aortic arch replacement: SVG to artificial aortic bypass (SVG-AO) or SVG to non-aortic bypass (SVG-non-AO). Which type is chosen is mainly according to the personal experience of surgeons. To prevent the artificial aorta from oozing blood, some doctors will wrap the aorta and implement a modified Cabrol fistula technique (6–9). However, as it is difficult to bypass SVG to the proximal aorta after wrapping, the secondary artery is used for proximal artery bypass grafting in most patients with STAAD concomitant with CABG (SVG-non-AO). Overall, some doctors suggest that the rate of resurgery has become lower with the improvement of meticulous care and precise techniques and the effect of wrapping the aorta and the modified Cabrol fistula technique may not be significant (10, 11); thus, bypass can be performed on the artificial aorta without wrapping the aorta. Nevertheless, the prognosis of patients undergoing these two approaches is unclear. Indeed, both may have adverse effects on grafts. On the one hand, the aortic wall of the artificial artery is more rigid than the normal aorta, which may lead to problems related to the proximal anastomosis of the SVG. On the other hand, the non-aorta (brachiocephalic trunk, left common carotid artery, and left subclavian artery) flow is lower than that in the aorta, and low flow is associated with graft occlusion (12).

Therefore, our study aimed to clarify the early- and mid-term prognosis of these two groups of patients and to provide a reference for the choice of the location of the proximal end of the graft in STAAD concomitant with CABG.

## Materials and Methods

### Patients

**Figure 1:**
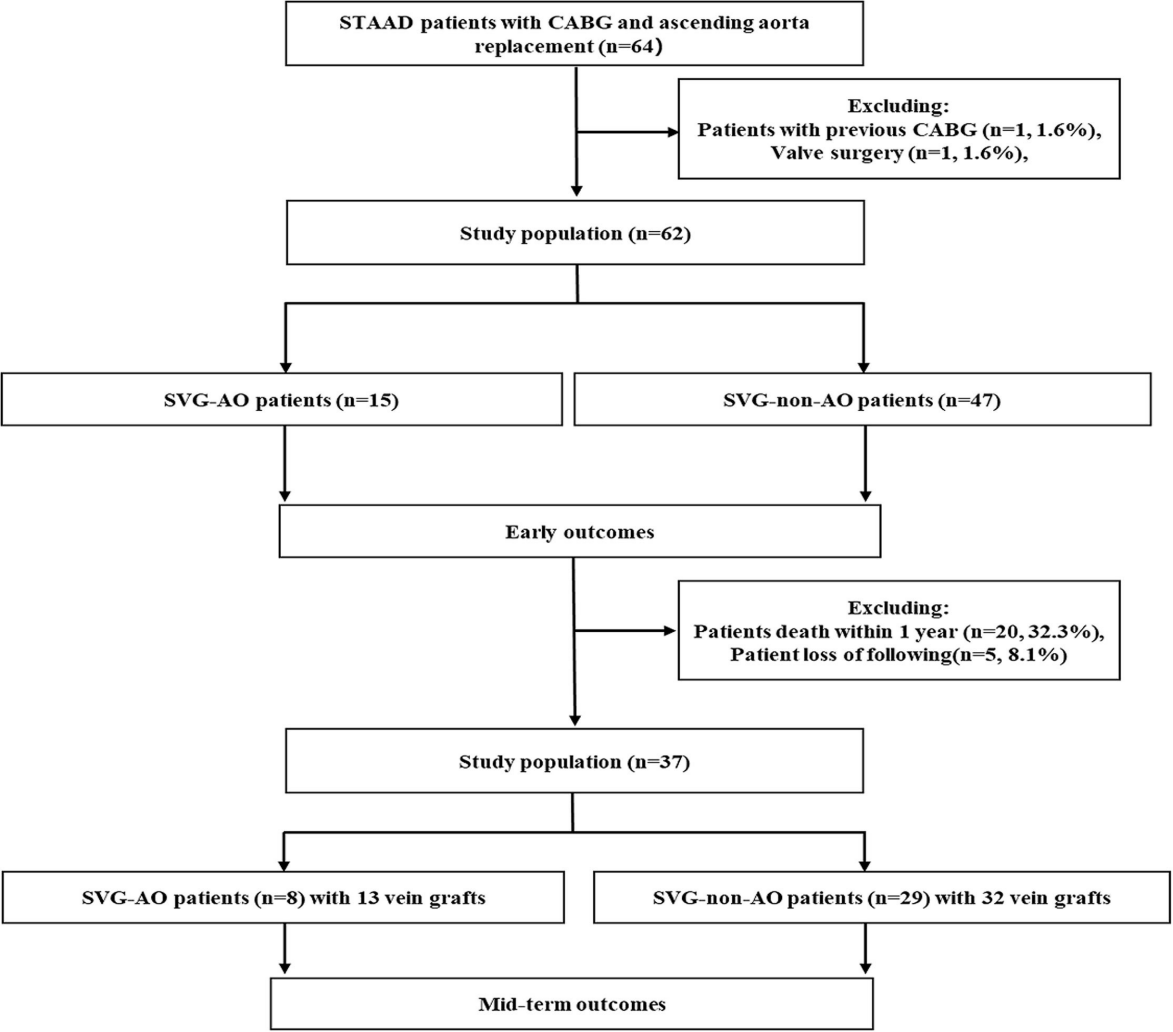
Screening process.

Inclusion criteria: from May 2015 to April 2020, patients with STAAD over 18 years old who received ascending aorta replacement + total aortic arch + frozen elephant trunk surgery concomitant with CABG at our hospital were analyzed retrospectively. Exclusion criteria: patients with previous CABG surgery (*n* = 1) and previous valve surgery (*n* = 1). Ultimately, 62 patients were enrolled in our study (Figure 1). The protocol of this retrospective study was approved by the Anzhen Hospital Ethics Committee. Because the study did not involve the specific personal information of the patient, the Ethics Committee waived the need for informed consent from each patient.

### Surgical Procedure

All patients were given ascending and total aortic arch replacement, and frozen elephant trunk stenting, cardiopulmonary bypass, circulatory arrest, and selective cerebral perfusion were used during the surgery. All patients underwent CABG because of coronary malperfusion or previous coronary artery disease (some patients experienced sudden myocardial infarction during the surgery). The proximal bypass sites included the artificial aorta, left subclavian artery, left common carotid artery, and right brachiocephalic artery. The great saphenous vein was used as the graft in all patients. In some patients, sandwich sutures were used for SVG-AO (Supplementary Figure 1). Considering the risk of STAAD, aortic replacement would be performed first before CABG. The acquisition of internal mammary artery may delay the process of aortic surgery. Furthermore, some patients have bilateral subclavian artery involvement. It is unclear whether the acquisition of radial artery aggravates the degree of upper limb blood shortage. Therefore, we choose the most stable bypass graft—the great saphenous vein to ensure the safety of patients in our study.

### Definitions and Endpoints

**Figure 2:**
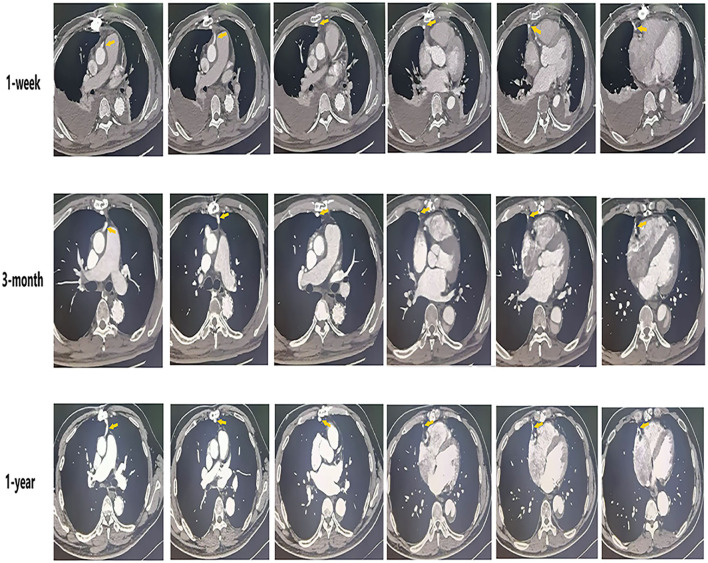
The condition of SVG-AO grafts at different times (coronary CT angiography).

**Figure 3:**
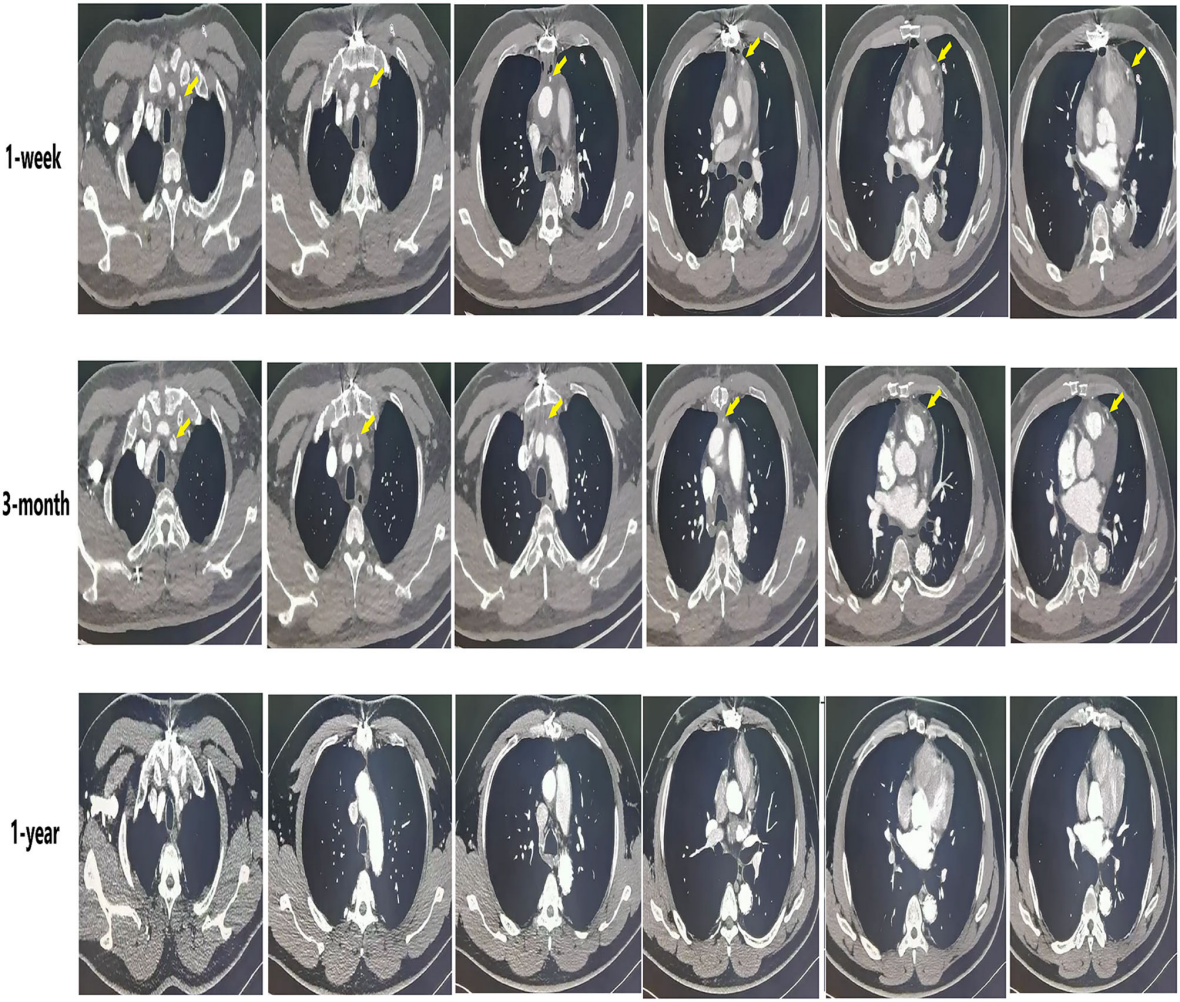
The condition of SVG-non-AO grafts at different times (coronary CT angiography).

Circulatory arrest time was defined as the time from the start of cardiopulmonary bypass arrest to the recovery of lower limb circulation. Ascending aorta occlusion time was defined as the time from blocking the ascending aortic circulation to recovery the ascending aortic circulation after the establishment of cardiopulmonary bypass. SVG-AO is defined as the proximal end of the SVG bypass to the aorta (Figure 2). SVG-non-AO is defined as the proximal end of SVG bypass to the left subclavian artery (Figure 3), the left common carotid artery or the right brachiocephalic artery. According to the Fitzgibbon classification, grade A (graft stenosis <50%) is considered a patent graft and grade B a stenosis graft (graft stenosis >50%); grade C indicates that the graft cannot be defined as grade A or grade B, and grade U denotes complete graft occlusion. Early outcomes are death and bleeding. Bleeding was defined as blood loss <200 ml/h within 3 h after the surgery. Mid-term outcomes were graft patency and aortic-related events. Aortic-related events included aortic resurgery, aortic rupture, or new aortic dissection.

### Statistical Analysis

For a normal distribution, an independent sample *t*-test was performed for comparisons of continuous variables between the two groups. Continuous variables that did not conform to a normal distribution were analyzed by the Mann–Whitney *U*-test. The Pearson's chi-squared test and Fisher's precision probability test were applied for comparisons of frequency variables. Univariate risk factors for early survival were compared with Kaplan–Meier curves and log-rank tests between the two groups. Cox regression analysis was employed to determine independent risk factors for early death, and logistic analysis was implemented for univariate and multivariate analyses of 1-year graft occlusion. Variables with *p* < 0.20 in univariate analysis were included in multivariate regression analysis. A two-tailed *p* < 0.05 was considered statistically significant. We used IBM SPSS Statistics version 26 (IBM Corp., Armonk, NY, USA) for all of the above analyses.

## Results

### Baseline

The mean age of the patients in our study was 56.7 years, with no significant difference between the two groups (Table 1); 80.6% of the patients were male. The SVG-AO group had a significantly longer time from symptom onset to surgery (*p* = 0.039) and a higher rate of previous coronary artery disease (*p* = 0.032). Additionally, there was no significant difference in body mass index (26.1 vs. 26.0 kg/m^2^, *p* = 0.521), body surface area (1.85 vs. 1.9 m^2^, *p* = 0.516), previous hypertension (100 vs. 85.1%, *p* = 0.180), previous diabetes (26.7 vs. 6.4%, *p* = 0.052), previous cerebral infarction (6.7 vs. 10.6%, *p* = 1.000), smoking (53.3 vs. 63.8%, *p* = 0.541), previous oral antithrombotic medicines (13.3 vs. 17.0%, *p* = 1.000), previous oral lipid-lowering medicines (0 vs. 10.6%, *p* = 0.323), previous oral antihypertensive medicines (6.7 vs. 6.4%, *p* = 1.000), NYHA classification (*p* = 0.443), previous cardiac surgery (26.7 vs. 12.8%, *p* = 0.237), preoperative coronary malperfusion (EF20 vs. 9%, *p* = 0.443), or previous cardiac surgery (26.7% vs. 12,4926.2) between the two groups.

**Table 1:** Baseline of different proximal target arteries of SVG in STAAD^a^.

**Variable**	**All patients**	**SVG-AO^**b**^ bypass**	**SVG-non-AO^**c**^ bypass**	***p*-value**
	***n* = 62**	***n* = 15 (24.2)**	***n* = 47 (75.8)**	
Age (years), mean ± SD	56.7 ± 9.8	58.8 ± 12.5	56.1 ± 8.8	0.349
Male, *n* (%)	50 (80.6)	12 (80.0)	38 (80.9)	1.000
BMI (kg/m^2^)^d^, medium (IQR)	26.1 (3.4)	26.1 (3.6)	26.0 (3.2)	0.521
BSA^e^, mean ± SD	1.87 ± 0.28	1.85 ± 0.40	1.9 ± 0.27	0.516
Time from symptom onsets to surgery (hours), medium (IQR)	5.5 (20)	12 (48)	4 (20)	0.039^*^
Coronary artery disease, *n* (%)	28 (45.2)	13 (86.7)	25 (53.2)	0.032^*^
Hypertension, *n* (%)	55 (88.7)	15 (100)	40 (85.1)	0.180
Diabetes, *n* (%)	7 (46.7)	4 (26.7)	3 (6.4)	0.052
Cerebral infarction, *n* (%)	6 (9.7)	1 (6.7)	5 (10.6)	1.000
Smoking, *n* (%)	38 (61.3)	8 (53.3)	30 (63.8)	0.541
Oral anti-thrombotic medicines, *n* (%)	10 (16.1)	2 (13.3)	8 (17.0)	1.000
Oral lipid lowering medicines, *n* (%)	5 (8.1)	0 (0)	5 (10.6)	0.323
Oral antihypertensive medicines, *n* (%)	4 (6.5)	1 (6.7)	3 (6.4)	1.000
NYHA classification^f^, *n* (%)				0.443
I	45 (72.6)	12 (80.0)	33 (70.2)	
II	11 (17.7)	2 (13.3)	9 (19.1)	
III	5 (8.1)	1 (6.7)	4 (8.5)	
IV	1 (1.6)	0 (0)	1 (2,1)	
Previous history of cardiac surgery, *n* (%)	10 (16.1)	4 (26.7)	6 (12.8)	0.237
Renal malperfusion, *n* (%)	12 (19.4)	2 (13.3)	10 (21.3)	0.713
Coronary malperfusion, *n* (%)	10 (16.1)	3 (20.0)	7 (14.9)	0.693
EF (%) ^g^, medium (IQR)	60 (7)	60 (8)	60 (6)	0.492

a *Great saphenous vein graft and Stanford type A aortic dissection*.

b *The proximal end of the great saphenous vein graft bypassed the aorta*.

c *The proximal end of great saphenous vein graft bypassed to a secondary artery*.

d *Body mass index*.

e *Body surface area*.

f *New York Heart Association*.

g *Ejection fraction*.

* *p < 0.05*.

### Perioperative and Post-operative Outcomes

As shown in Table 2, the mean surgery time in all patients was 9 h, and there was no significant difference between the groups (7.5 vs. 9 h, *p* = 0.166). Moreover, the cardiopulmonary bypass time (221 vs. 236 min, *p* = 0.593), ascending aorta occlusion time (130 vs. 117 min, *p* = 0.480), and cardiac arrest time (30.0 vs. 24.3 min, *p* = 0.061) were not significantly different between the two groups. However, unexpected emergency CABG was less common in the SVG-AO group (26.7 vs. 44.7%), though the difference was not statistically significant (*p* = 0.245). There also were no significant differences in blood transfusion during surgery (53.3 vs. 48.9%, *p* = 1.000) or the minimum anal temperature (25.2 vs. 25.8°C, *p* = 0.217) or minimum nasal temperature (24.1 vs. 24.5°C, *p* = 0.300) between the two groups. Regarding early post-operative outcomes, there was no significant difference in drainage volume at 24 h after the surgery (475 vs. 630 ml, *p* = 0.766), post-operative neurological complications (6.7 vs. 10.6%, *p* = 1.000), post-operative paraplegia (6.7 vs. 4.3%, *p* = 1.000), post-operative arrhythmia (26.7 vs. 23.4%, *p* = 1.000), post-operative myocardial infarction (13.3 vs. 2.1%, *p* = 0.143), post-operative ARDS (26.7 vs. 23.4%, *p* = 1.000), post-operative stroke (0 vs. 2.1%, *p* = 1.000), post-operative low cardiac output (33.3 vs. 23.4%, *p* = 0.505), or early resurgery (13.3 vs. 14.9%, *p* = 1.000). Post-operative acute kidney injury (13.3 vs. 27.7%, *p* = 0.322) and dialysis (6.7 vs. 21.3%, *p* = 0.268) were less common in the SVG-AO group, but the difference was not significant.

**Table 2:** Perioperative and early post-operative outcomes of different proximal target arteries of SVG in STAAD^a^.

**Variable**	**Overall**	**SVG-AO bypass^**b**^**	**SVG-non-AO bypass^**c**^**	***p*-value**
	***n* = 62**	***n* = 15 (24.2)**	***n* = 47 (75.8)**	
Surgery time (h), medium (IQR)	9 (3)	7.5 (4)	9 (3)	0.166
CPB time (min)^d^, medium (IQR)	235.5 (101)	221 (164)	236 (100)	0.593
Cardiac arrest time (min)^e^, mean ± SD	25.7 ± 10.2	30.0 ± 12.6	24.3 ± 9.0	0.061
Ascending aorta occlusion time (min)^f^, medium (IQR)	118.5 (52)	130 (46)	117 (53)	0.480
Unexpected emergency coronary bypass^g^, *n* (%)	25 (40.3)	4 (26.7)	21 (44.7)	0.245
Blood transfusions in surgery, *n* (%)	31 (50.0)	8 (53.3)	23 (48.9)	1.000
Minimum anal temperature (°C), medium (IQR)	25.7 (1.7)	25.2 (1.3)	25.8 (1.8)	0.217
Minimum nasal temperature (°C), medium (IQR)	24.2 (1.5)	24.1 (1.8)	24.5 (1.4)	0.300
Post-operative neurological complications, *n* (%)	6 (9.7)	1 (6.7)	5 (10.6)	1.000
Post-operative paraplegia, *n* (%)	3 (4.8)	1 (6.7)	2 (4.3)	1.000
Post-operative arrhythmia, *n* (%)	15 (24.2)	4 (26.7)	11 (23.4)	1.000
Post-operative myocardial infarction, *n* (%)	3 (4.8)	2 (13.3)	1 (2.1)	0.143
Post-operative acute kidney injury, *n* (%)	15 (24.2)	2 (13.3)	13 (27.7)	0.322
Post-operative dialysis, *n* (%)	11 (17.7)	1 (6.7)	10 (21.3)	0.268
Drainage volume 24 h after surgery (ml), medium (IQR)	605 (615)^h^	475 (547.5)^h^	630 (655)^h^	0.766
Post-operative blood transfusion>3 U, *n* (%)	33 (56.9)^h^	4 (33.3)^h^	29 (63.0)^h^	0.101
Post-operative ARDS^i^, *n* (%)	15 (24.2)	4 (26.7)	11 (23.4)	1.000
Post-operative stroke, *n* (%)	1 (1.6)	0 (0)	1 (2.1)	1.000
ICU time (day)^j^, medium (IQR)	2 (4)	2 (1)	2 (4)	0.064
Early resurgery^k^, *n* (%)	9 (14.5)	2 (13.3)	7 (14.9)	1.000
Post-operative low cardiac output, *n* (%)	16 (25.8)	5 (33.3)	11 (23.4)	0.505
Death, *n* (%)	20 (32.3)	6 (40.0)	14 (29.8)	0.532

a *Great saphenous vein graft and Stanford type A aortic dissection*.

b *The proximal end of the great saphenous vein graft bypasses the aorta*.

c *The proximal end of the great saphenous vein graft bypassed to a secondary artery*.

d *Cardiopulmonary bypass time*.

e *Circulatory arrest time was defined as the time from the start of cardiopulmonary bypass arrest to the recovery of lower limb circulation*.

f *Ascending aorta occlusion time was defined as the time from blocking the ascending aortic circulation to recovery the ascending aortic circulation after the establishment of cardiopulmonary bypass*.

g *An urgent need for coronary artery bypass grafting during the surgery, which was not planned before the surgery*.

h *Four patients were excluded because of death immediately after the surgery*.

i *Acute respiratory distress syndrome*.

j *Intensive Care Unit time*.

k *Early post-operative secondary surgery resulting from bleeding*.

### Early Outcomes

**Figure 4:**
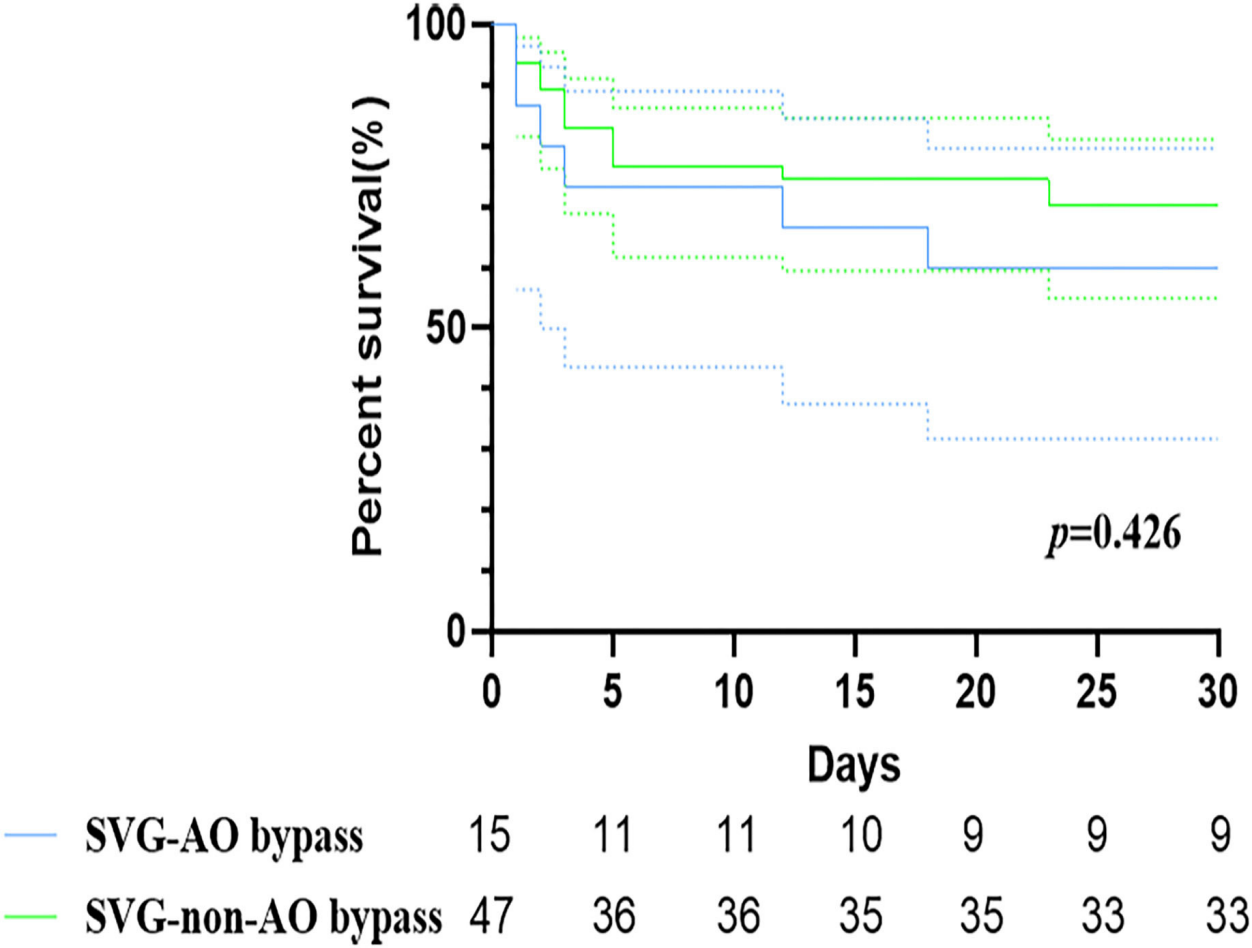
Kaplan–Meier curves of SVG-AO and SVG-non-AO grafts within 30 days. Blue line: The great saphenous vein bypassed the aorta. Green line: The great saphenous vein bypassed to a secondary artery.

Early mortality did not differ significantly between the two groups [six patients (40.0%) vs. 14 patients (29.8%), *p* = 1.000, Table 2, Figure 4]. Six patients in the SVG-AO group died, and 14 died within 30 days in the hospital. Thirteen patients died due to heart failure, three due to respiratory failure, three due to multiple organ dysfunction syndromes, and one due to cerebral infarction. According to the log-rank test, the hazard ratio of early mortality was 1.459 (SVG-AO/SVG-non-AO), and the 95% CI was 0.5124 to 4.152, without statistical significance (*p* = 0.426). The drainage volume at 24 h after the surgery was 475 ml in the SVG-AO group and 630 ml in the SVG-non-AO group, without a significant difference (*p* = 0.766). As shown in Table 3, there were a total of 80 grafts. The main distal sites of CABG were the anterior descending artery (30 grafts with 37.5%) and the right coronary artery (42 grafts with 52.5%). The locations of the proximal arteries of SVG-non-AO were the left subclavian artery (*n* = 18), left common carotid artery (*n* = 19), and right brachiocephalic artery (*n* = 19).

**Table 3:** Proximal and distal of coronary artery bypass grafting.

**Distal bypass location**	**All grafts**	**SVG-AO bypass^**a**^**	**SVG-non-AO bypass** ^**b**^		
	***n* = 80**	**Aorta**	**Left subclavian artery**	**Left common carotid artery**	**Right brachiocephalic artery**
		***n* = 24**	***n* = 18**	***n* = 19**	***n* = 19**
LAD^c^	30 (37.5)	11 (45.8)	5 (27.8)	10 (52.6)	4 (21.1)
RCA^d^	42 (52.5)	9 (37.5)	10 (55.6)	8 (42.1)	15 (78.9)
LCX^e^	5 (6.25)	4 (16.7)	1 (5.6)	0 (0)	0 (0)
MA^f^	1 (1.25)	0 (0)	0 (0)	1 (5.3)	0 (0)
OA^g^	2 (2.5)	0 (0)	2 (11.1)	0 (0)	0 (0)

a *The proximal end of the great saphenous vein graft bypassed the aorta*.

b *The proximal end of the great saphenous vein graft bypassed to a secondary artery*.

c *The left anterior descending coronary artery*.

d *The right coronary artery*.

e *The left circumflex coronary artery*.

f *The middle coronary artery*.

g *The opposite coronary artery*.

### Mid-term Outcomes

The median follow-up was 33.5 months. As shown in Table 4, the patency graft of all patients was 46/48 (95.8%) at 1 week after surgery, among which one graft had complete occlusion and one had stenosis (>50%). About 15 (93.3%) grafts were patent in the SVG-AO group and 32/33 (97%) grafts in the SVG-non-AO group. There was no significant difference between the two groups at 1 week after the surgery. At 3 months after the surgery, 28/45 (62.2%, one patient with one graft was lost to follow-up) grafts were patent; 12/13 (92.3%) grafts were patent in the SVG-AO group and 16/32 (50%) grafts in the SVG-non-AO group, with a significant difference (*p* = 0.015). At 1 year after the surgery, 27/45 (60%) grafts were patent in all patients. About 12/13 (92.3%) grafts were patent in the SVG-AO group, 15/32 (46.9%) grafts were patent in the SVG-non-AO group, with a significant difference between the two groups at 1 year (*p* = 0.006). Univariate analysis showed that SVG-AO, age, statins, and Clopidogrel + Aspirin were protective factors against graft failure (Table 5), whereas BMI was a risk factor; SVG-AO was an independent protective factor for graft failure, and BMI was an independent risk factor. As shown in Table 6, no patients underwent aortic-related resurgery at 1 year. Three patients had bleeding-related complications within 1 year (two patients had an oral mucosal hemorrhage, and one had cerebral hemorrhage). All three patients took Clopidogrel + Aspirin. There was no significant difference in the use of anticoagulants or lipid-lowering drugs between the SVG-AO group and the SVG-non-AO group. Although there was a significant difference in graft patency between the two groups, there was no significant difference in coronary artery disease-related symptoms.

**Table 4:** Fitzgibbon patency classification of patients with frozen elephant trunk surgery concomitant with CABG^a^.

	**All patients**	**SVG-AO group^**b**^**	**SVG-non-AO group^**c**^**	***p*-value**
	**No (%)**	**No (%)**	**No (%)**	
1-week graft patency	*n* = 48 grafts (40 patients)	*n* = 15 grafts (10 patients^e^)	*n* = 33 grafts (30 patients^f^)	0.532
Fitzgibbon classification^d^				
Grade A	46 (95.8)	14 (93.3)	32 (97.0)	
Grade B	1 (2.1)	0 (0)	1 (3.0)	
Grade U	0 (0)	0 (0)	0 (0)	
Grade O	1 (2.1)	1 (6.7)	0 (0)	
3-month graft patency	*n* = 45 grafts (37 patients)	*n* = 13 grafts (8 patients^g^)	*n* = 32 grafts (29 patients^h^)	0.015^*^
Fitzgibbon classification				
Grade A	28 (62.2)	12 (92.3)	16 (50.0)	
Grade B	10 (22.2)	0 (0)	10 (31.3)	
Grade U	1 (2.2)	0 (0)	1 (3.1)	
Grade O	6 (13.3)	1 (7.7)	5 (15.6)	
1-year graft patency	*n* = 45 grafts (37 patients)	*n* = 13 grafts (8 patients)	*n* = 32 grafts (29 patients)	0.006^*^
Fitzgibbon classification				
Grade A	27 (60.0)	12 (92.3)	15 (46.9)	
Grade B	6 (13.3)	0 (0)	6 (18.8)	
Grade U	0 (0)	0 (0)	0 (0)	
Grade O	12 (26.7)	1 (7.7)	11 (34.3)	

a *Coronary artery bypass grafting surgery*.

b *The proximal end of the great saphenous vein graft bypassed the aorta*.

c *The proximal end of the great saphenous vein graft bypassed the secondary artery*.

d *Fatzgibbon classification: A, Widely patent graft (stenosis <50%); B, Patent graft but with a flow-limiting lesion; U, A patent graft but unable to differentiate between A and B owing to poor coronary CT angiography; O: An occluded graft*;

e *One patient without post-operative coronary artery CT angiography*.

f *Four patients without post-operative coronary artery CT angiography*.

g *Two patients died within 3 months*.

h *One patient died within 3 months*,

* *: Grade B, Grade U, and Grade O were classified as non-patency; Grade A vs. Grade B + Grade U + Grade O, p < 0.05*.

**Table 5:** Logistic regression of graft failure at a year after surgery.

**Univariate regression analysis**				**Multivariate regression analysis**			
**Variables**	**OR^**a**^**	**95% CI^**b**^**	***p*-value**	**Variables**	**OR^a^**	**95% CI^**b**^**	***p*-value**
SVG-AO^c^	0.065	0.008–0.560	0.013^*^	SVG-AO^c^	0.009	0.000–0.520	0.023^*^
BMI^d^	1.528	1.115–2.093	0.008^*^	BMI^d^	1.938	1.171–3.206	0.010^*^
Age	0.937	0.874–1.004	0.065	Age	0.915	0.892–1.108	0.994
Statins^e^	0.427	0.110–1.657	0.219	Statins^e^	0.299	0.045–2.000	0.213
Clopidogrel + Asprin^f^	0.229	0.060–0.878	0.032	Clopidogrel + Asprin^f^	0.206	0.034–1.258	0.087

a *Odds ratio*.

b *95% CI*.

c *The proximal end of the great saphenous vein graft bypassed the aorta*.

d *Body mass index*.

e *Post-operative oral statins*.

f *Dual anticoagulant therapy within 1 year after surgery*.

* *p < 0.05*.

**Table 6:** Mid-term outcomes of patients with STAAD concomitant with CABG^a^.

**Variable**	**All patients**	**SVG-AO bypass^**b**^**	**SVG-non-AO bypass^**c**^**	***p*-value**
	***n* = 37**	***n* = 8 (23.1)**	***n* = 29 (76.9)**	
Aortic-related reoperation	0 (0)	0 (0)	0 (0)	1.000
Oral mucosal hemorrhage	2 (5.4)	0 (0)	2 (6.9)	1.000
cerebral hemorrhage	1 (2.7)	0 (0)	1 (3.4)	1.000
Clopidogrel + Aspirin	13 (35.1)	3 (37.5)	10 (34.5)	1.000
Statins	10 (27.0)	2 (25.0)	8 (27.6)	1.000
Symptoms of coronary heart disease	7 (18.9)	2 (25.0)	5 (17.2)	0.653

a *Coronary artery bypass grafting*.

b *The proximal end of the great saphenous vein graft bypassed the aorta*.

c *The proximal end of the great saphenous vein graft bypassed to a secondary artery*.

## Discussion

Aortic dissection is sometimes accompanied by coronary artery problems (13, 14). Although some serious coronary artery problems require CABG during surgery, there is some controversy regarding the location of the proximal end of the bypass. For type A aortic dissection, the ascending aorta will be replaced with an artificial aorta. However, as the patency of SVG bypass to the artificial aorta is not clear, some doctors choose a secondary artery, such as the left subclavian artery, as the location of the proximal bypass. Our study compared early- and mid-term results between these two groups. We found no significant difference in terms of the early patency of the graft or early outcomes. Nevertheless, SVG bypass to the secondary artery did have poor graft patency in the mid-term (3 months and 1 year) compared to SVG bypass to an artificial aorta. There were no aortic-related events in either group at the 1-year follow-up.

The STAAD with concomitant CABG is associated with high operative mortality (15, 16), in accordance with our study (32.3% early mortality in all CABG patients in our study). CABG is recommended for some patients with serious previous coronary artery disease or coronary artery involvement with type B or type C according to Neri's classification (3, 17), though this situation will have some problems. On the one hand, some patients have severe myocardial ischaemia or even myocardial infarction before surgery, and early coronary reperfusion is necessary. There is also a risk of sudden rupture of the aorta. The contradiction between them (priority coronary reperfusion and aortic dissection) may be one of the main reasons. Aortic dissection is sometimes accompanied by coronary artery problems (13, 14). Although some serious coronary artery problems require CABG during surgery, there is some controversy regarding the location of the proximal end of the bypass. For type A aortic dissection, the ascending aorta will be replaced with an artificial aorta. However, as the patency of SVG bypass to the artificial aorta is not clear, some doctors choose a secondary artery, such as the left subclavian artery, as the location of the proximal bypass. Our study compared early- and mid-term results between these two groups. We found no significant difference in terms of the early patency of the graft or early outcomes. Nevertheless, SVG bypass to the secondary artery did have poor graft patency in the mid-term (3 months and 1 year) compared to SVG bypass to an artificial aorta. There were no aortic-related events in either group at the 1-year follow-up.

The STAAD with concomitant CABG is associated with high early mortality. A small number of patients received priority CABG or distal retrograde perfusion of cardioplegia. Due to ascending aortic dissection, the proximal bypass location of the SVG can only use the secondary artery without dissection. On the other hand, Zhang et al. (9) reported that wrapping the aorta and presenting a modified Cabrol fistula technique can significantly reduce post-operative bleeding in STAAD. Nonetheless, proximal aortic bypass after this method is difficult, and some doctors choose a secondary artery for SVG proximal bypass. In our study, there was no significant difference in 24-h drainage volume between the two groups. The reason may be that in the aortic bypass group, aorta wrapping shunts and sandwich sutures of artificial vessel patch SVGs were used for some patients. With regard to early results, there was no significant difference in our study between different proximal bypass methods for early death and bleeding.

The patients with CABG were followed up for 1 week, 3 months, and 1 year. At 3 months and 1 year, the graft failure of SVG-non-AO was significantly higher than that of SVG-AO, and the latter was an independent protective factor for 1-year graft patency. We consider the main reason to be the fact that the aortic flow is higher than that of a secondary artery, indirectly increasing the flow of grafts. In addition, the angle of the anastomosis may affect the coronary circulation (18), and blood flow of 70 to 90° was highest. The angle of the aorta may be closer to the best angle, though the angle of the secondary artery may not. In addition, some reports show that the use of double antiplatelets and statins after surgery is beneficial for vein graft patency (19, 20). However, there was no significant difference in post-operative antiplatelet and statin levels between the groups. Post-operative double antiplatelet therapy was a protective factor for 1-year graft patency; some patients with STAAD have a residual dissection of the abdominal aorta, and dual antiplatelet therapy may increase the risk of aortic-related events. The number of patients using dual antiplatelet therapy in our study was 13. Except for two patients with oral mucosal hemorrhage and one patient with cerebral hemorrhage, there were no aortic-related events, but larger sample size is needed for verification. In addition, there was no significant difference in coronary heart disease-related symptoms between the two groups at the 1-year follow-up, which may be due to the formation of collateral circulation. In conclusion, our mid-term results suggest that SVG-AO should be the first choice of proximal bypass location for patients with STAAD and bypass grafts and that dual antiplatelet therapy should be used as much as possible after surgery if there is no risk of bleeding. These 62 patients did not undergo coronary angiography before the operation because the situation was urgent and a coronary angiogram might aggravate dissection. After surgery, most patients did not have symptoms of coronary ischemia, there were only seven patients (18.9%) who had symptoms of coronary artery disease. In these patients, only two patients received coronary angiograms. Both patients belonged to the SVG-non-AO group, and the angiographic results showed that the grafts were occluded.

Our study also has some limitations. First, at baseline, the time from symptom onset to surgery and previous coronary artery disease was significantly different between the two groups, which may be due to the selection bias of retrospective studies. Second, because STAAD cases with CABG are very rare, our sample size was relatively small, which may have caused some bias. Although coronary artery angiography is the gold standard to evaluate the coronary artery, it is an invasive examination, and there may be a risk of aortic adverse events with coronary angiography in some patients with residual abdominal aortic dissection. Therefore, we used coronary computed tomography angiography to evaluate the graft, which may lead to inaccurate evaluation of some coronary stenoses.

## Data Availability Statement

The raw data supporting the conclusions of this article will be made available by the authors with the consent of the authors.

## Ethics Statement

The studies involving human participants were reviewed and approved by the Anzhen Hospital Ethics Committee. Written informed consent was not required for this study, in accordance with the local legislation and institutional requirements.

## Author Contributions

HZ and MW designed the whole conception. HZ, MG, and LS provided the administrative support. MW, SJ, and MG provided the data of patients. MW, SJ, and XP collected and assembled the data. MW was a major contributor in writing the manuscript. All authors have read and approved the final manuscript.

## Funding

This study was supported by the National Key R&D Program of China (2017YFC1308000), the National Science Foundation of China (81770466), and the Beijing Municipal Administration of Hospital's Ascent Plan (DFL20180602).

## Conflict of Interest

The authors declare that the research was conducted in the absence of any commercial or financial relationships that could be construed as a potential conflict of interest.

## Publisher's Note

All claims expressed in this article are solely those of the authors and do not necessarily represent those of their affiliated organizations, or those of the publisher, the editors and the reviewers. Any product that may be evaluated in this article, or claim that may be made by its manufacturer, is not guaranteed or endorsed by the publisher.

## Supplementary Material

Supplementary Figure 1 The great saphenous vein graft bypass to the aorta with wrapping the aorta.

## Abbreviations

STAAD: Stanford type A aortic dissection
CABG: Coronary artery bypass disease
SVG: The great saphenous vein
SVG-AO: The great saphenous vein bypassed to the aorta
SVG-non-AO: The great saphenous vein bypassed to a secondary artery
NYHA: New York Heart Association.
