# Supplementary figures and images for: Mid-term Patency of the Great Saphenous Bypass to Aorta vs. Non-aortic Arteries in Stanford Type A Aortic Dissection Surgery With Concomitant CABG
Source: Front Cardiovasc Med. 2021 Oct 26;8:743562. doi: 10.3389/fcvm.2021.743562 (PMC8576286; doi:10.3389/fcvm.2021.743562)

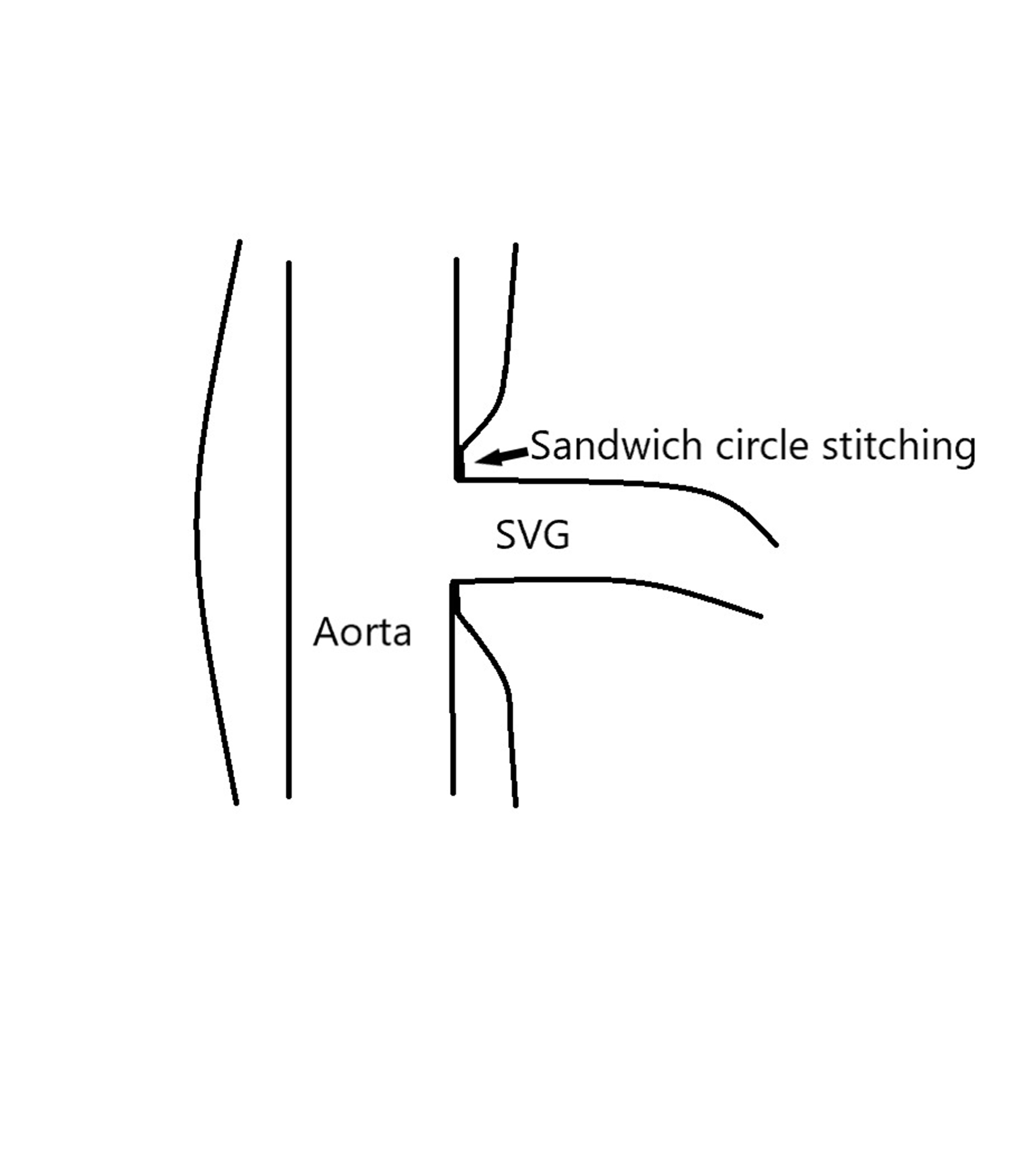

Supplement: Supplementary Figure 1 — The great saphenous vein graft bypass to the aorta with wrapping the aorta. [file Image_1.JPEG]
